# Within and beyond the boundaries of proteasomal assembly: PSMD9 chaperone as a multifunctional protein

**DOI:** 10.1042/EBC20253044

**Published:** 2026-02-23

**Authors:** Rangapriya Sundararajan, Mahalakshmi Harish, Merlyn Cherusserikkaran Anthony, Joel Christie, Damini Jaiswal, Prasanna Venkatraman

**Affiliations:** 1 Protein Interactome Lab for Structural and Functional Biology, Advanced Centre for Treatment, Research and Education in Cancer, Tata Memorial Centre, Navi Mumbai, Maharashtra, 410210, India; 2 Center for Cell and Gene Therapy, Texas Children’s Hospital, Baylor College of Medicine, Houston, Texas, 77030, U.S.A.; 3 Homi Bhabha National Institute, Mumbai, Maharashtra, 400094, India; 4 F. M. Kirby Neurobiology Center, Department of Neurobiology, Boston Children’s Hospital, Harvard Medical School, Boston, Massachusetts, 02115, U.S.A.

**Keywords:** assembly, cancer, chaperone, interactions, proteasome, PSMD9, SLiM

## Abstract

PSMD9/Nas2/Bridge-1 is one of the assembly chaperones of the 19S regulatory particle of the eukaryotic proteasome. While this is among PSMD9’s well-recognized roles, the role of PSMD9 in cancer proteasome assembly/disassembly and activity, as a key factor in the ubiquitination and degradation of proteins by the proteasome, unfolded protein response, and proteostasis, nucleolar organization, are some of the recent findings. Several unbiased screening, high-throughput studies, genome-wide association studies (GWAS) have found surprising associations and potential roles for PSMD9 in a variety of diseases or conditions. Although in a majority of these cases the mechanism remains unclear, it is important to take note that this multi-functional protein, in the absence of any enzymatic role, relies primarily on its ability to interact with other proteins and biomolecules in the cells. A surprising range of proteins that associate with PSMD9 discovered by the structure-guided approaches overlaps with many different functions associated with this protein or the proteasome in literature. Collective evidence also points to the possibility that PSMD9 could be an Achilles’ heel in some of the solid cancers.

## Introduction

Proteasomes and lysosomes represent key mechanisms for protein degradation in various biological systems [1–3]. Proteasomes degrade cellular proteins that are marked by ubiquitination as well as proteins that are unfolded or carry long intrinsically disordered regions that may not require ubiquitination. Inhibitors targeting the active sites of proteasomes are utilized in the treatment of multiple myeloma and, to a lesser extent, mantle cell lymphoma [4–6]. However, these same inhibitors are ineffective against solid tumors. Some of the proposed reasons include inadequate drug penetration, heterogeneity within tumors, activation of autophagy, and immune evasion [7]. Resistance to such 20S active site inhibitors in cells is also triggered by lower levels or depletion of 19S regulatory particle subunits of the proteasome [8,9], the mechanisms of which are unclear.

**Figure 1:**
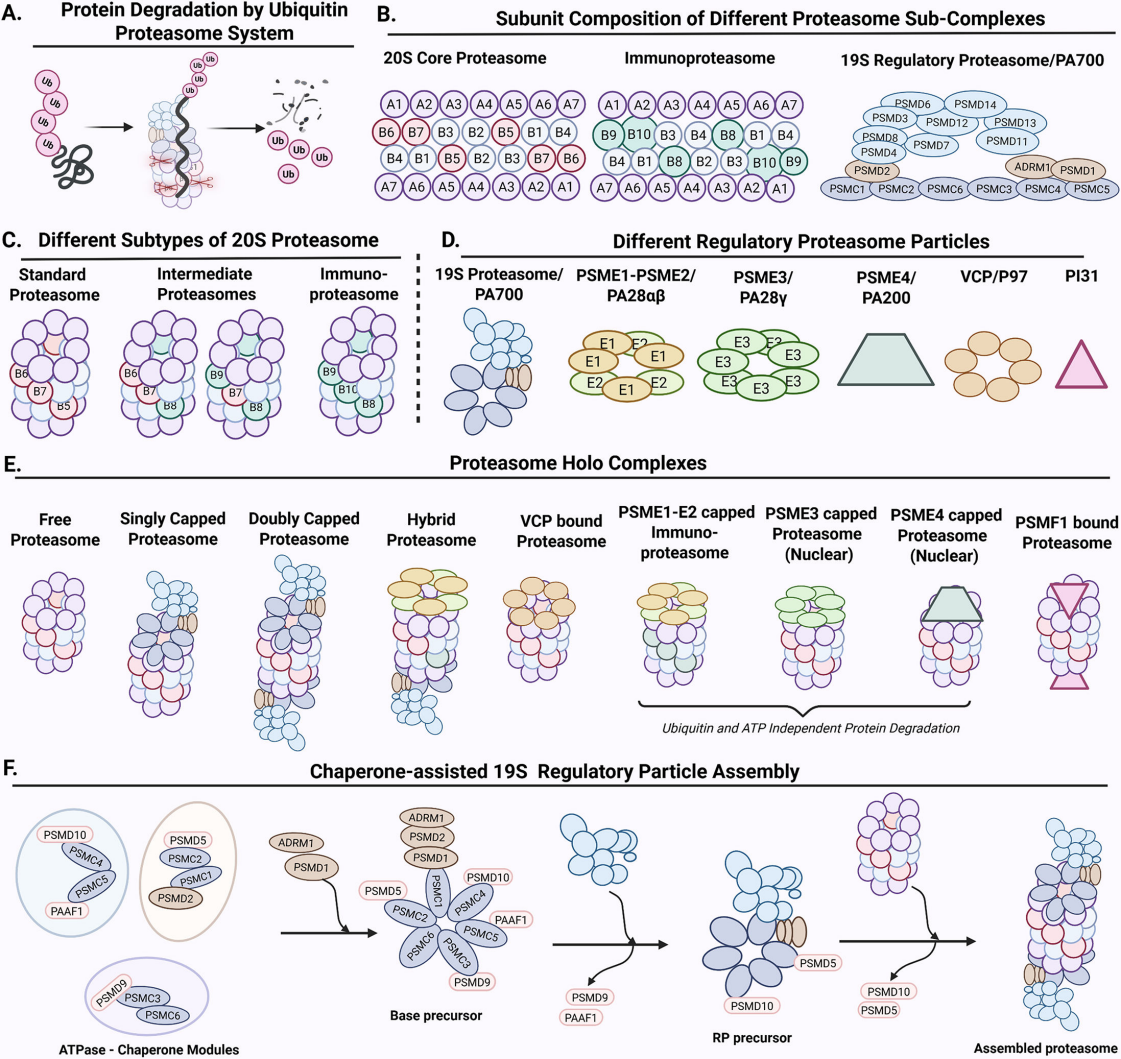
The structural and functional diversity of proteasome complexes in protein degradation

The proteasome’s minimal structural unit is the 20S core particle (CP) also called as 20S proteasome, capable of degrading unfolded proteins and peptides (Figure 1). This barrel-shaped structure consists of 28 subunits arranged in four stacked heptameric rings—two outer α-rings that form openings for substrate entry and two inner β-rings containing the catalytic sites PSMB5, PSMB6, and PSMB7, providing chymotrypsin-like, caspase-like, and trypsin-like activities, respectively [10–12]. Variations in subunit composition lead to diverse proteasome subtypes, contributing to the system’s structural and functional complexity (Figure 1). The constitutive proteasome, composed of the PSMB5, PSMB6, and PSMB7 subunits, predominates in most tissues, ensuring efficient protein quality control and turnover. Tumor cells often up-regulate constitutive subunits to meet the increasing metabolic needs, making them susceptible to active site inhibitors like bortezomib. In response to inflammatory signals such as interferon-γ or TNF-α, the immunoproteasome incorporates PSMB8, PSMB9, and PSMB10 subunits, modifying peptide generation for MHC class I presentation and enhancing immune responses [13]. Intermediate proteasomes, which combine both constitutive and immuno proteasome subunits, are found in specific cancers, producing antigenic peptides [14].

(**A**) A schematic of the ubiquitin proteasome system (UPS) illustrates the process by which the proteasome recognizes and degrades ubiquitinated proteins into short peptides. (**B**) The subunit composition of various proteasome subcomplexes. In the 20S core proteasome, there are α- and β-subunits; immunoproteasomes contain alternative catalytic β-subunits (PSMB8, PSMB9, PSMB10); and the 19S regulatory proteasome contains several ATPases (PSMC1-6) and non-ATPase (PSMDs) subunits. (**C**) Different 20S proteasome subtypes. Standard proteasomes, intermediate proteasomes, and immunoproteasomes exhibit different catalytic subunit compositions and cleavage preferences. (**D**) Regulatory proteasome particles. These comprise the 19S particle, PSME1, PSME2, PSME3, PSME4, VCP/p97, and PI31, which regulate substrate recognition, processing, or degradation. (**E**) The 20S proteasome forms can assemble with regulatory particles such as 19S, PSME1, PSME2, PSME3, PSME4, PA200, VCP/p97, or PI31. These holo complexes enable context-dependent degradation, either 26S proteasome (ubiquitin- and ATP-dependent) or independent pathways. (**F**) Chaperone-mediated 19S Regulatory particle assembly. PSMD9, PSMD10, PSMD5 and PAAF1 interact with ATPase subunits to form modules, which form base precursor by interacting with PSMD1 and ADRM1. The base precursor interacts with lid sub-complex to form 19S RP precursor which further interacts with 20S core to form fully assembled proteasome holo complexes.

The 20S proteasome capped at its ends by several forms of regulatory particles (RPs) adds to its structural and functional diversity. The 19S RP, a complex of about 700 kDa, consists of a base and a lid which can cap the 20S proteasome at one (26S) or both ends (30S) (Figure 1). Subunits in the lid recognize and bind to the ubiquitin tag on the substrates, the deubiquitinating enzymes remove polyubiquitin chains, and the hexametric ATPase ring made up of PSMC1-PSMC6 subunits at the base unfolds the protein in an ATP-dependent manner, allowing its translocation into the 20S core for degradation [15]. An unstructured region of approximately 40 amino acids is mandatory to initiate degradation of the substrates [16,17]. Other regulatory particles, including the 11S proteasome activators PA28αβ (a PSME1–PSME2 heteroheptamer) and PA28γ (a PSME3 homoheptamer), as well as PA200 (PSME4) (Figure 1), influence proteasome activity through ubiquitin-independent pathways. While PA28αβ is largely involved in immunoproteasome-mediated antigen processing, PA28γ primarily acts in the nucleus to control the degradation of proteins linked to cell cycle and stress, thereby connecting proteasome activity to cellular stress response pathways [18,19].

The 20S and the 19S proteasomes are assembled from a total of 32 polypeptides. The registry/position of the subunits in the fully assembled complex must be ensured for the assembled proteasome to be functional. Because no one subunit is identical to the other, assembly of 20S, or 19S is a nonstochastic process and involves dedicated chaperones. Chaperones are proteins that either alone or by transient association with other proteins, facilitate proper folding (unfolding), assembly, or stabilization, without becoming a permanent structural component. Dedicated chaperones, considered also as proteasome subunits, assist in the formation of the 20S and the organization of the 19S RP complex [20–22]. For the nomenclature of the subunits and their functions, please refer to Table 1.

**Table 1:** 26S Proteasome subunit composition and auxiliary factors

Subunit type	Subunit name/Alias	Gene symbol	Subcomplex	Major function/Notes
20S Core Particle (CP)	α1	PSMA1	α-ring	Structural; forms gate of proteolytic chamber
	α2	PSMA2	α-ring	Structural scaffold
	α3	PSMA3	α-ring	Interacts with regulatory particles
	α4	PSMA4	α-ring	Ring stabilization
	α5	PSMA5	α-ring	Interacts with β subunits
	α6	PSMA6	α-ring	Gate regulation; docking site for 19S
	α7	PSMA7	α-ring	Terminal subunit; gate control
	β1	PSMB6	β-ring	Caspase-like activity (acidic residues)
	β2	PSMB7	β-ring	Trypsin-like activity (basic residues)
	β3	PSMB3	β-ring	Structural; assembly
	β4	PSMB2	β-ring	Structural; assembly
	β5	PSMB5	β-ring	Chymotrypsin-like activity (hydrophobic residues)
	β6	PSMB1	β-ring	Structural
	β7	PSMB4	β-ring	Essential for core dimerization
Immunoproteasome	β1i (LMP2)	PSMB9	β-ring	Replaces β1; antigenic peptide generation
	β2i (MECL-1)	PSMB10	β-ring	Replaces β2; alters trypsin-like activity
	β5i (LMP7)	PSMB8	β-ring	Replaces β5; modifies chymotrypsin-like activity
19S Regulatory Particle (RP) - Base (ATPases)	Rpt1	PSMC2	Base	ATPase; substrate unfolding
	Rpt2	PSMC1	Base	ATPase; translocation of substrates
	Rpt3	PSMC4	Base	ATPase; assists gate opening
	Rpt4	PSMC6	Base	ATPase; structural stability
	Rpt5	PSMC3	Base	ATPase; regulates gate opening
	Rpt6	PSMC5	Base	ATPase; substrate recognition
19S Regulatory Particle (RP) - Base (Non-ATPases)	Rpn2	PSMD1	Base	Scaffold; substrate docking
	Rpn1	PSMD2	Base	Ubiquitin-binding; receptor for Rpn10/13
	Rpn10 (S5a)	PSMD4	Base	Polyubiquitin receptor
19S Regulatory Particle (RP) - Lid	Rpn3	PSMD3	Lid	Structural; connects Rpn7 and Rpn8
	Rpn7	PSMD6	Lid	Structural; lid architecture
	Rpn8	PSMD7	Lid	With Rpn11 forms DUB module
	Rpn6	PSMD11	Lid–Base interface	Stabilizes 26S complex
	Rpn5	PSMD12	Lid	Structural scaffold
	Rpn9	PSMD13	Lid	Structural stability
	Rpn11	PSMD14	Lid	Metalloprotease DUB; removes ubiquitin chains
19S associated/Assembly Factors	PSMD5	PSMD5	Regulator	Negative regulator of 26S assembly
	PSMD8	PSMD8	Assembly factor	Proteasome assembly modulator
	PSMD9	PSMD9	Chaperone	Facilitates 19S assembly
	PSMD10 (Gankyrin)	PSMD10	Regulator	Oncogenic; interacts with CDK4 and MDM2
Other 19S Components	Rpn13	ADRM1	Base	Ubiquitin receptor; recruits UCH37
	Rpn15	Sem1 (DSS1)	Lid/Base interface	Small acidic protein; assembly factor
Alternative activators	PA28α	PSME1	Cytosol	IFN-γ–induced activator of 20S
	PA28β	PSME2	Cytosol	Partner with PA28α
	PA28γ	PSME3	Nucleus	ATP-independent nuclear activator
	PA200	PSME4	Nucleus	DNA repair and spermatogenesis
Assembly chaperones	POMP	POMP	20S	Proteasome maturation protein
	PAC1	PSMG1	α-ring	Chaperone for α-ring assembly
	PAC2	PSMG2	α-ring	Partners with PAC1
	PAC3	PSMG3	β-ring	β-ring assembly
	PAC4	PSMG4	β-ring	Partners with PAC3
Deubiquitinases	Rpn11	PSMD14	Lid	Essential DUB; Cleaves ubiquitin before degradation
	UCH37	UCHL5	Base	Reversible substrate trimming
	USP14	USP14	Base	Delays degradation; checkpoint role
Regulatory factor	PI31	PSMF1	Regulator	Negative regulator of 20S proteasome

Four primary chaperones are involved in 19S RP assembly, PSMD9, PSMD10, PSMD5, and PAAF1, each with unique structural and functional characteristics (Figure 1F). Each of these chaperones bring in two ATPases (PSMD9-PSMC3-PSMC6; PSMD5-PSMC2-PSMC1; PSMD10-PSMC4-PSMC5) which ultimately form the six-membered ATPase-ring at the base of the proteasome. In the final assembled 26S proteasome, the ATPases are arranged in a trimer-of-dimers configuration (PSMC1-PSMC2, PSMC6-PSMC3, PSMC4-PSMC5) (Figure 1F). Such a configuration of trimer-of-dimers is conserved in the hexameric ATPase ring of the Archaeal proteasomes called the PAN, as well as in the yeast proteasome. Unlike the eukaryotic counterparts, the PAN ATPase is made of a single chain that homo-oligomerises to form the six-membered ring, which serves the same function as in the eukaryotic proteasomes: gate opening, substrate unfolding, and translocation of the substrates [23], requiring an unstructured region of ~ 40 amino acids [24]. While the six eukaryotic ATPases show structural conservation, they differ substantially in their sequence, and none of them are stable when expressed alone. The interaction with chaperones probably stabilizes these ATPases, facilitating their binding and folding. Such a chaperone requirement is not reported for PAN.

Structural studies of proteasome assembly chaperones, namely, PSMD10 (ankyrin repeats), PSMD5 (armadillo/HEAT-like repeats), PSMD14 (WD40 domain), and PSMD9 (PDZ-like domain) demonstrated that, although their folds are distinct, they interact with specific C-terminal regions of the ATPase subunits with a clear distinction in the case of PSMD9: it binds to the same C-terminal residues in Rpt5 (PSMC3) that are important for interaction with the α-ring on 20S proteasome. Chaperone binding sterically inhibits the interaction of ATPase tails with the core particle alpha-ring. During final assembly, the C-terminal HbYX motifs of PSMC2, PSMC3, and PSMC5, each originating from a different dimer, interact with designated α-ring pockets, establishing stabilizing salt bridges that facilitate gate opening and induce chaperone release, thereby linking the trimer-of-dimers organization to the activation of the proteasome [21,25] (Figure 1). Roles of these functional chaperones in 19S proteasome assembly have been discussed extensively elsewhere [21,25]. In this review, we focus specifically on PSMD9, its role in and beyond proteasome assembly.

PSMD9 functions are largely deduced from associations derived from exploratory and unbiased screening approaches, such as high-throughput analyses and genome-wide association studies. A subset of PSMD9 functions is deduced from correlation studies that highlight direct or inverse relationships between PSMD9 and associated functions in various diseases and conditions. For instance, PSMD9 is linked to increased risks of adiposity, type 2 diabetes, cardiovascular diseases, and schizophrenia [26–28]. Furthermore, PSMD9 is implicated in therapy resistance in cervical and breast cancers [29,30] and promotes malignant tumor progression in hepatocellular carcinoma [31]. PSMD9 is one of the genes that is sensitive to nutlin (a small molecule inhibitor of p53), response in liposarcoma cell lines. It has also been identified as a regulator of lipid metabolism in rodents [32]. In the *Drosophila* glial cells, PSMD9 was found critical for the regulation of proteasomal activity and exosome formation; inhibition of which could curb the high-sugar diet (HSD) induced neuropathy symptom [33]. In the same paper, under HSD or diabetic-like conditions, PSMD9 was found to act as a regulator of diabetes-associated peripheral neuropathy, and down-regulation of PSMD9 alleviates these symptoms. In neurodegenerative diseases like Parkinson’s, PSMD9 is among the proteasomal subunits that are overexpressed [34]. These correlative lines of evidence implicate that PSMD9 is up-regulated and is associated with age-associated disorders such as cancers, diabetes, and various neurological disorders. While the exact mechanisms are largely unclear, PSMD9 seems to be up-regulated as an adaptive response to cellular stress such as proteotoxic stress, tumorigenicity, aberrant cellular signaling, and organelle-specific protein quality control (PQC) burden.

Proteins consisting of separate, flexibly connected domains are frequently repurposed across diverse biological contexts through the engagement of different interaction partners. Such proteins also function across various cellular states by engaging alternative binding partners via a common interaction interface, as exemplified by tumor suppressors and oncoproteins [35]. PSMD9 aligns with this paradigm: it comprises two distinct domains-N and PDZ, a configuration commonly linked to functional adaptability. PDZ domains in other systems function as modular scaffolds that identify a variety of protein and lipid partners via short linear motifs (SLiM) of about 4–10 contiguous stretches of amino acids, thereby facilitating swift reconfiguration of interaction networks and cellular processes. Consistent with this behavior, the PDZ domain of Bridge-1, the rat ortholog of PSMD9, interacts with the transcriptional coactivator p300 to modulate insulin gene expression, demonstrating that PSMD9 or its orthologs can engage in non-proteasomal signaling pathways [36]. Although PSMD9 (Nas2 in yeast) is conserved as a 19S assembly chaperone, the significant sequence divergence between yeast and mammalian homologs indicates that PSMD9 may have gained additional interactions and biological roles in higher eukaryotes. It potentially acts as a context-dependent interaction hub, connecting proteasome biogenesis with wider cellular pathways. Systematic mapping of PSMD9 protein–protein interactions, employing affinity purification–mass spectrometry, co-expression network-level analyses, and motif-guided discovery, thus constitutes a robust approach to identify unexpected functions that go beyond traditional proteasome biology.

Our own efforts over the years have established that the seemingly innocuous assembly chaperone, PSMD9, is an important player in the pathophysiology of solid cancers. Based on the literature evidence so far, this review covers what we know about PSMD9 in the context of proteasome assembly and other cellular functions, with emphasis on cancer, and argues that this chaperone could be an Achilles heel, at least in breast cancer.

### PSMD9 impacts holo-complex structure in a context-dependent manner

In order to obtain insights into the cancer proteasome structure, assembly, heterogeneity, and functions, we used knowledge from the fundamental rules of protein interactions: to assemble the unit proteasome consisting of subunits with distinct sequences and functions, the interacting partners must be coexpressed and present at least in stoichiometric amounts in cells. Proteasomal subunits in breast tumor tissues exhibited a progressive reduction in the correlated mRNA and protein expression with increasing disease severity. This motivated us to reconstruct a structural model of the patient breast tumor proteasome using the available EM structures weighted with expression correlations. In addition, since all pairwise subunit RNA correlations were used, it was also possible to identify regulators of the assembly which are not part of the final proteasome structure, such as chaperones. PSMD9 emerged as a key assembly regulator, negatively impacting the formation of 26S and 30S proteasomes [37]. While PSMD9 is known to promote base assembly towards holocomplex formation in mammalian cells, the PSMD9 knockout in MCF7 breast cancer cells unexpectedly increased the levels and activities of both forms of the proteasome holocomplexes, 26S and the 30S, that degrade ubiquitinated proteins. This trend was also reported in another study in mammalian cells [38]. Incidentally, while depletion of another 19S assembly chaperone PSMD5/s5b enhanced 26S/30S holo-complex levels, PSMD10 depletion reduced 30S levels. Decreased PSMD5/s5b levels in the PSMD9 knockout or increased PSMD9 levels in the PSMD10 knockout were proposed as mechanisms to explain the 26S/30S increase or 30S decrease, respectively. Therefore, at least in mammalian cells, the assembly chaperones appear to compensate for each other’s absence and distinctly impact the final ratios of the different forms of the proteasome [38].

In mammals, PSMD9 recruits PSMC3 and PSMC6 ATPases by directly binding PSMC3. This conserved tri-modular complex associates with other 19S regulatory particle assembly intermediates (Figure 1). PSMD9 detaches before 19S formation [21]. During proteasome assembly in MCF7 breast cancer cells triggered by bortezomib exposure, the PSMC3 ATPase was found to integrate into the 26S and 30S proteasome independent of PSMD9. Surprisingly, PSMC6 ATPase failed to incorporate efficiently in the absence of PSMD9. Along similar lines, an ATP hydrolysis mutant of Rpt4 (yeast PSMC6 homolog), *rpt4-EQ,* was more sensitive to the deletion of nas2 (yeast PSMD9 ortholog) than the *rpt5-EQ* mutant (yeast PSMC3 ortholog) in terms of cell growth and holo-proteasome activity [39]. Appropriate loading of the paired ATPase subunits PSMC3-PSMC6 into assembly intermediates and the final 26S/30S proteasome, therefore, is dependent on PSMD9. In the absence of PSMD9, low incorporation of PSMC6 could potentially create structural ‘gaps’ in the proteasome, facilitating the formation of alternative proteasome variants. Alternative proteasomes containing the duplicated PSMA7 subunit [40] or the immunoproteasome are known to form during adaptive survival response to proteotoxic stress. Furthermore, PSMD9 knockout enhanced the accumulation of polyubiquitinated proteins in MCF7 cells. A combination of diverse factors, such as functional impairment of the deregulated breast cancer proteasome, altered shuttle factors, and/or altered substrate influx, could potentially impact ubiquitin-mediated proteolysis in PSMD9-knockout cells.

### Role of PSMD9 in the formation of assembly intermediates and quality control

Breast cancer cell lines such as MCF7 and MDAMB231 tend to accumulate 19S assembly intermediates compared with the MCF10A breast normal-like control cells. In addition to perturbing the 19 S-20S interface and holo-complex structures, PSMD9 knockout significantly enhanced the recovery of proteasomal assembly intermediates in MCF7(37). Depletion of another assembly chaperone, PSMD5, in cell lines and mouse tissues also led to assembly intermediate structures containing the 19S base complex and 20S harboring some of the 19S subunits [38]. Recent evidence points to the possibility of these assembly intermediates attracting quality control mechanisms. For instance, the HERC1 E3-ubiquitin ligase acts as a quality control of proteasome assembly in MCF7 cells by suppressing the accumulation of PSMC5:PAAF1-containing assembly intermediates [41]. Furthermore, aberrant assembly intermediates are spatially restricted to the nucleus as part of quality control [42]. More recently, studies in yeast have pinpointed Nas2 as a potential proteasome assembly checkpoint acting through steric hindrance in the penultimate step of base assembly and delaying the formation of the complete base. Upon formation of the correct 19S base, Rpt4 hydrolysis serves as a signal for Nas2 release [39]. Structural studies also show that Nas2 shields the Rpt1-binding site on Rpt5, preventing premature base assembly [43]. Thus, Nas2 acts as a checkpoint regulator or a licensing factor of 19S assembly and 20S and 19S association. By analogy, PSMD9 may serve similar roles in mammalian cells, and its loss (PSMD9 knockout) can lead to premature assembly and activation that can be detrimental, as seen in the luminal breast cancer cell lines.

### Potential role of PSMD9 in 20S homeostasis

An unanticipated outcome of network analysis of the patient breast tumor proteasome was the significant functional connectivity of PSMD9 with 20S-PSMA and 20S-PSMB subunits, i.e., Pearson correlation coefficient *r* > 0.4 in normal breast luminal tissues which was reduced in tumors [37]. PSMD9 depletion in MCF7 resulted in a significant reduction in the PSMA7 20S-α subunit levels which is required for free 20S formation [44]. Additionally, the β5/PSMB5 catalytic subunit retains functional connectivity with PSMD9 in patient luminal tumor tissues (Pearson correlation *r* = 0.73 in breast normal vs *r* = 0.49 in luminal tumors, *P*=0.02). In this context, Bortezomib treatment led to a partial but significant defect in PSMB5-mediated chymotrypsin-like activity in luminal breast cancer MCF7 cells lacking PSMD9. Both proteotoxic and oxidative stress induce proteasomal subunit expression and de novo proteasome biogenesis. Upon bortezomib treatment, aside from a transient PSMC3 integration defect, PSMD9-depleted MCF7 cells did not show any other anomaly that could fully explain the activity decrease. Neither NRF2 expression, which is known to induce β5/PSMB5 expression and activation under stress [45], nor proteasome biogenesis was affected. These correlations raise the possibility of PSMD9 impacting 20S homeostasis, the precise mechanisms of which are currently unknown.

### PSMD9 couples tumor proteasome structure to stress adaptation and survival pathways in breast cancer (BrCa) homeostasis

Under normal conditions, overexpressed PSMD9 in MCF7 breast cancer cells correlates with a 20S-High state, and its loss reduces free 20S levels and induces a 26S/30S (ratio)-High state [37]. PSMD9 presence promotes cell cycle progression by facilitating PSMA7 and CCNE1 expression while reducing the p21/CiP1 CDK inhibitor levels. The accumulation of polyubiquitinated proteins and elevated NRF2 in PSMD9-depleted cells signifies proteotoxic and oxidative stress, which leads to G1/S growth arrest and reduced anchorage-independent growth, indicating impaired tumorigenicity [37]. Interestingly, the unstructured p21 is a substrate of the 20S proteasome [46], and PSMD9 may enhance the degradation of certain CDK inhibitors such as p21, thereby enabling continued cell proliferation even in the presence of substantial proteotoxic or oxidative damage. The 20S high state, which correlates with a high level of PSMD9 in MCF7 cells compared with the MCF10A transformed but non-malignant breast epithelial cell line, potentially contributes to tumorigenicity by such mechanisms. Thus, PSMD9 depletion is a potentially clinically relevant strategy to simultaneously impair proteasome function and regulate the stability of select proteins in solid tumors that are resistant to 20S-targeted therapies.

### Role of PSMD9 in aberrant cellular signaling

PSMD9 appears to be responsive to TGF-β signaling in breast carcinoma and human granulosa cell lines which up-regulate both PSMD9 and SMAD proteins [47]. Through its interaction with the p300 protein via the PDZ domain, the PSMD9 ortholog, rat Bridge-1, functions as a coactivator of insulin gene expression [36], and overexpression of Bridge-1 in pancreatic β-cells leads to diabetes mellitus in rats [48] driven by apoptotic death of the islets.

**Figure 2:**
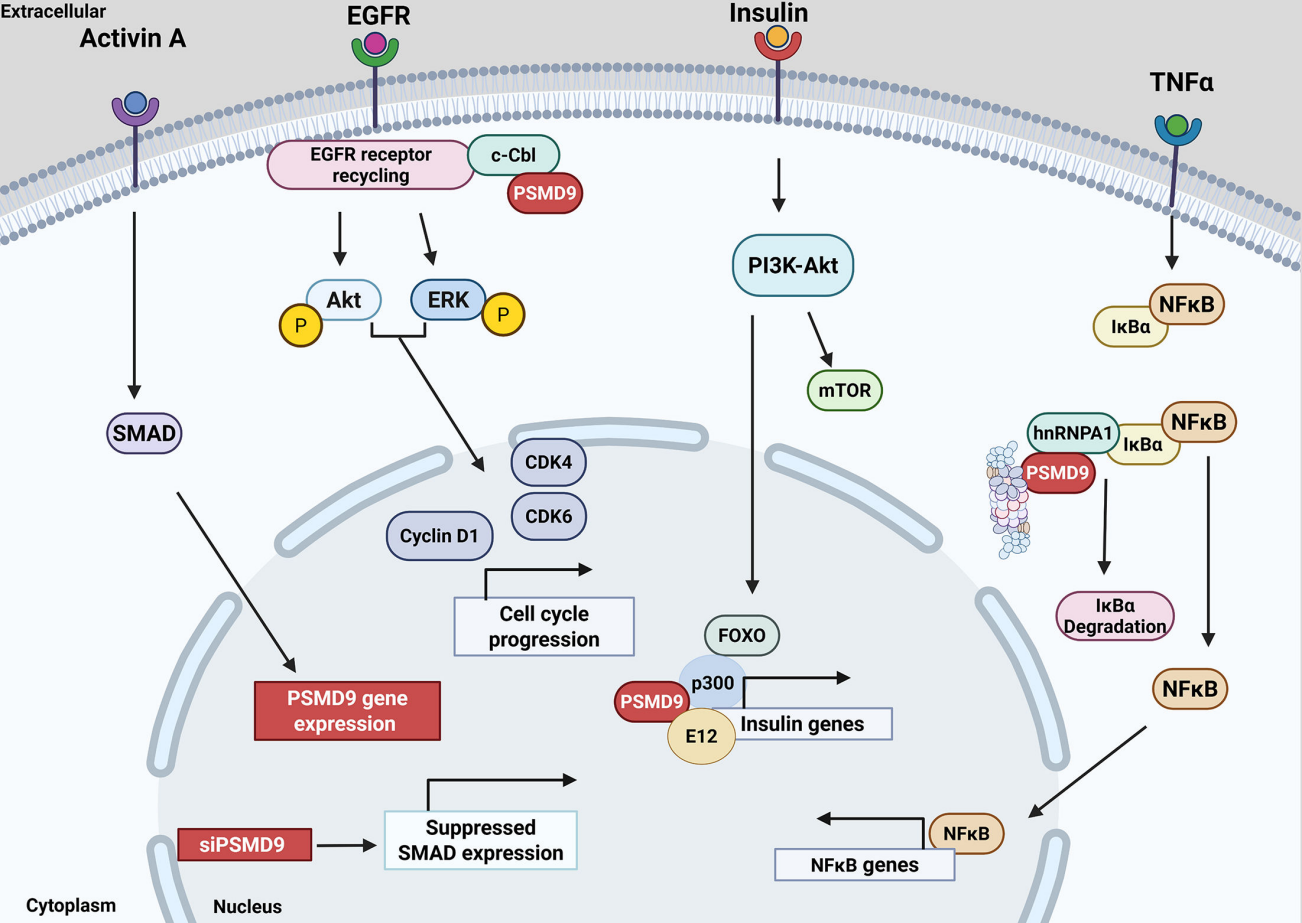
The Role of PSMD9 in cellular signaling.

Furthermore, PSMD9 facilitates the progression of hepatocellular carcinoma (HCC) by interacting with c-Cbl, an E3 ubiquitin ligase, which prevents the proteasomal degradation of the Epidermal Growth Factor Receptor (EGFR). This interaction results in the stabilization and sustained activation of EGFR, promoting its recycling and subsequently activating oncogenic signaling cascades such as ERK1/2 and Akt pathways [31]. PSMD9 enhances NF-κB signaling by facilitating the proteasomal degradation of IκBα through a specialized adaptor-like mechanism [49]. PSMD9 interacts with hnRNPA1, forming a complex through a C-terminal PDZ-binding motif. This interaction promotes the effective recruitment of IκBα to the 26S proteasome. This enhances IκBα turnover, relieving the inhibition on NF-κB and facilitating the nuclear translocation of the p65 subunit. Consequently, NF-κB–dependent transcription is elevated. Disruption of PSMD9 or its interaction with hnRNPA1 stabilizes IκBα and reduces NF-κB activation, demonstrating that PSMD9 acts as a selective proteasomal regulator that connects protein–protein interactions to growth, oncogenic, and inflammatory signaling pathways (Figure 2).

PSMD9 influences pathways such as NF-κBα, insulin, cell cycle, and TGF-β signaling. In TGF-β signalling, activation of the pathway using Activin A leads to an increase in SMADS as well as PSMD9. In EGFR signalling, PSMD9 interacts with c-Cbl, influencing EGFR recycling, activating Akt/ERK/Cyclins/CDKs, and promoting cell cycle progression. PSMD9 also interacts with transcriptional coactivator p300 and E12, transcriptionally regulating insulin signalling. PSMD9 activates NF-κB signalling in PSMD9 overexpressing cells, by interaction with hnRNPA1, which interacts with ubiquitinated IκBα, targeting IκBα for proteasomal degradation, and thereby freeing NF-κB.

One of the important unifying outcomes of the studies on domain interaction of PSMD9 in mammalian cells and Bridge-1 in rat is as follows: the D157P mutation in PSMD9 impairs its interaction with hnRNPA1 and NF-κB activation, while the corresponding D156P mutation in Bridge-1 disrupts interaction with p300, a histone acetyl transferase, and inhibits insulin gene transcription. These point mutations are in the PDZ domain, and their effect is seen in cells that are transfected with the mutant protein. Bridge-1 also binds to E12, a basic helix-loop-helix containing protein, and deletion of nine residues of E12 inhibits interaction with Bridge-1 and affects insulin biogenesis. Human E12 also interacts with PSMD9, and mutation of the last residue Met or the C-terminal deletions inhibits interaction. AGHM, the four residues of human E12 bind to PSMD9 and the corresponding sequence in rat is AGHL. From the available reports, it looks like Bridge-1 acts as a multi-protein connector, stabilizing the transcriptional complex by binding to p300, E12, and PDX1 and enhancing glucose response in insulin regulation [36,50]. It is interesting to note that Bridge-1 can interact with internal regions in p300 and with N-terminal region in PDX 1, raising the possibility that PSMD9 can also act like such a scaffold which can orient protein complexes on transcriptional sites or in signaling cascades through the PDZ domain, not to mention the interactions made by N domain of PSMD9.

### Organelle dynamics and structure

PSMD9 is critical for preserving nucleolar architecture through the interaction with unbound ribosomal proteins (RPs) and modulating their trafficking and retention within the nucleolus [51]. The loss of PSMD9 perturbs nucleolar morphology, resulting in irregular and decondensed nucleoli, as well as mislocalization of NPM1, and induces nucleolar stress. Notably, re-expression of PSMD9, but not PSMD10, in the KO cells rescues the nucleolar morphology (unpublished data). This nucleolar stress subsequently triggers the release of ribosomal proteins such as RPS25 and RPL15 into the nucleoplasm. These free RPs stabilize WT p53 by inhibiting MDM2. As a result, p53 degradation is impeded, leading to an extended p53 half-life, which subsequently induces cell cycle arrest and diminished viability. Therefore, PSMD9 indirectly sustains basal p53 levels by ensuring nucleolar integrity and inhibiting RP-mediated MDM2 suppression.

**Figure 3:**
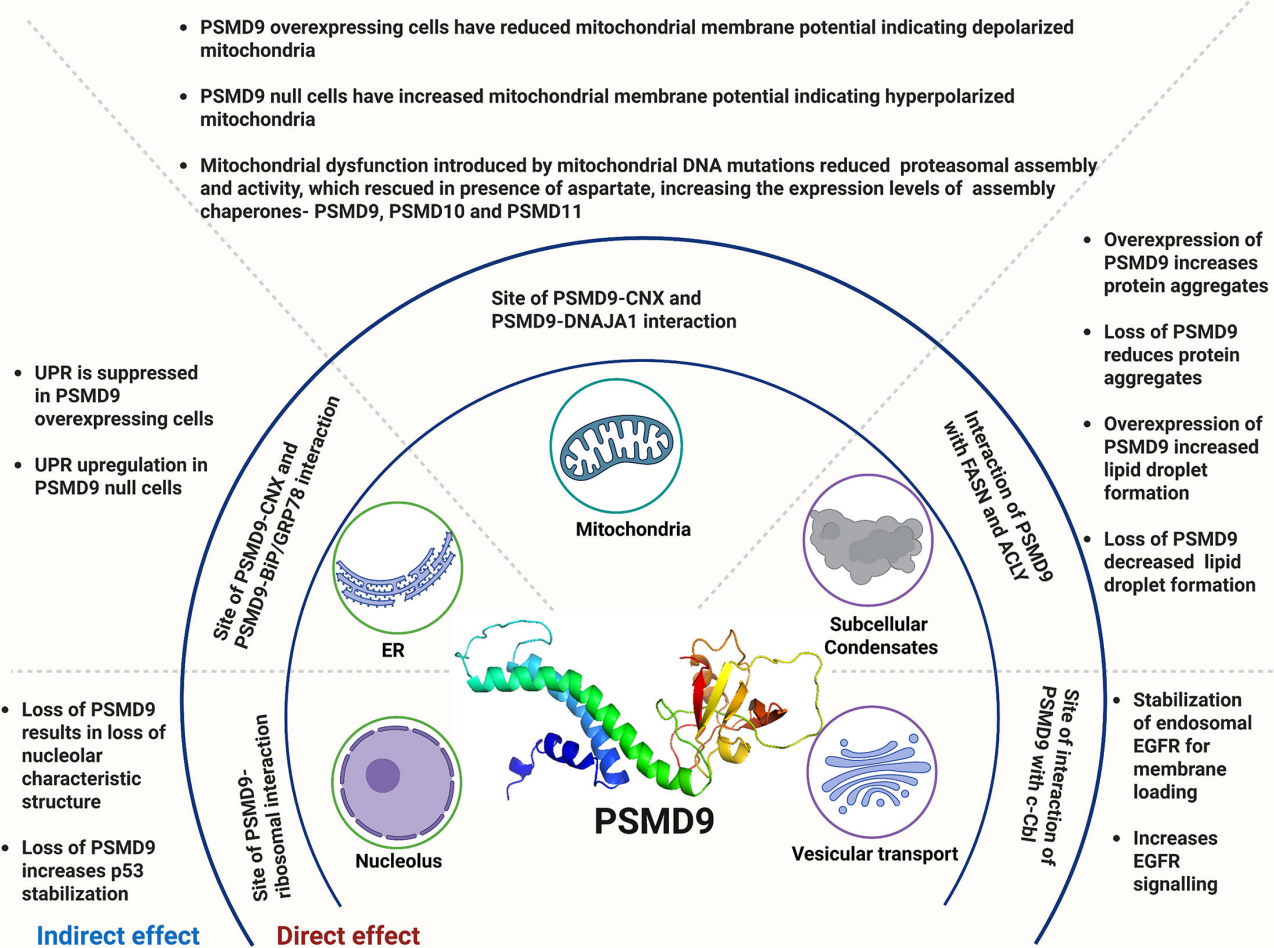
PSMD9 modulates organelle structure, dynamics, and functions

At the mitochondria-associated membrane, PSMD9 targets the mitochondrial co-chaperone DNAJA1 for proteasomal degradation presumably by directly interacting with it via an ELKK motif in the DNAJA1 J-domain as seen using purified proteins [52]. DNAJA1 stability in cells is limited by this interaction since the PSMD9–DNAJA1 complex is stabilized and DNAJA1 levels are raised when the proteasome is inhibited. The functional outcome of PSMD9 overexpression is depolarization of the mitochondrial membrane, while PSMD9 deletion causes hyperpolarization of the mitochondria. These membrane potential alterations imply that PSMD9 may affect mitochondrial dynamics, possibly redistributing the balance of fusion, fission, and mitophagy and adjusting mitochondrial function to cellular stress conditions. One of the mechanisms could be the indirect effect of PSMD9 on mitochondrial protein import and homeostasis by PSMD9 interaction mediated regulation of DNAJA1 turnover. Calnexin, a mitochondrial-associated membrane protein involved in ER-Mitochondrial Contact Sites (ERMCS) [53,54], interacts with PSMD9, indicating that PSMD9 may directly regulate ER (unpublished data) and mitochondrial structure and function [52]. These observations indicate that PSMD9 has a more general role in organelle homeostasis, although the precise mechanism requires more detailed and thorough mechanistic studies (Figure 3).

PSMD9 overexpression in HEK293 or as seen in cancer cells impacts the nucleolar architecture, ER, and mitochondrial function, protein aggregates, and lipid droplets. Loss of PSMD9 by CRISPR knockout reverses the observed phenotypes. In HCC, PSMD9 influences endosomal transport of EGFR. Direct effect: Physical interaction and the site of interaction represent the proposed domain-motif interface. Indirect effect: The mechanism is not clear, but some of the phenotypes can be rescued by overexpressing PSMD9 domains or full-length proteins in the PSMD9 KO background.

### PSMD9 buffers proteostasis

Chaperone machinery plays a crucial role in protein quality control, preventing aggregation and mitigating UPR. While proteasomal ATPases can unfold proteins, their uncoupling from degradation can exacerbate protein aggregation and cellular stress. We showed that PSMD9 overexpression in HEK293 cells increased lipid droplet formation while knockout led to its decrease (Figure 3). Re-expression of PSMD9 increases droplet abundance and mitigates UPR, potentially through interactions with fatty acid synthase (FASN) and BiP/GRP78 [55]. Lipid droplets serve as buffers for aggregation-prone proteins and frequently co-localize with aggresomes, suggestive of their role in proteostasis and lipid metabolism [56–58], a mechanism conserved in models of neurodegeneration and cancer [59,60]. In conditions of proteotoxic stress, including mitochondrial protein import overload or denervation, PSMD9 expression is up-regulated as a component of the adaptive proteasome remodeling program, aligning with NRF1-dependent ‘bounce-back’ regulation [61,62]. In addition, as seen from our studies, PSMD9 knockout triggered ER stress and up-regulated UPR markers (sXBP1, CHOP). PSMD9’s putative direct interactions with BIP/GRP78 (a UPR regulator) and FASN (a lipid metabolism enzyme) in cells associate the chaperone with these stress mitigation pathways. Besides the re-expression of the full-length protein, the N-terminal domain or the PDZ domain in PSMD9 KO cells rescued lipid droplet formation. The full-length PSMD9 restored lipid droplet levels to those of control cells, while the interaction-deficient mutant was less efficient [55]. Thus, PSMD9 functions as a proteostatic buffer, integrating signals from lipid metabolism, protein translation, folding, and degradation to maintain homeostasis. These mechanism positions PSMD9 as a central or one of the prominent hubs within the proteostasis network and a promising therapeutic target for modulating cellular stress responses and protein aggregation-related diseases (Figure 3).

### Clinical relevance of PSMD9 and its role in therapy resistance

Clinical and experimental data support an involvement of PSMD9 in mediating radioresistance across various cancers, including breast [29], cervical [30], and prostate [63]. High PSMD9 expression correlates with increased local recurrence and radioresistance, while low expression in breast cancer correlates with reduced recurrence in irradiated patients. Functional studies show PSMD9 knockdown enhances radiosensitivity in breast cancer cells [29].

While PSMD9 is a promising predictive biomarker for radiation response, its radioresistance mechanisms remain unexplored. Protein-protein interaction studies in HEK293 cells reveal that PSMD9 interacts with fatty acid synthase (FASN) [55]. Notably, FASN inhibition has independently been shown to enhance radiation-induced cell death in colorectal cancer models, suggesting a potential mechanistic link [64]. PSMD9 also promotes IκBα degradation through hnRNPA1 interaction, activating NF-κB, a pathway known for cancer survival and therapy resistance [49]. Furthermore, radiation induces NRF1-mediated up-regulation of proteasome subunits, including PSMD9, leading to DEPTOR degradation and elevated mTORC1 signaling, a hallmark of radioresistance [65]. These findings raise the possibility of multiple links between PSMD9 and radioresistance, pending further validation.

Beyond radiotherapy, emerging data indicate that PSMD9 may influence tumor responses to other forms of therapy-induced stress. In glioblastoma (GBM), PSMD9 expression increases with tumor grade and is associated with poor overall survival [66]. Experimental validation shows that PSMD9 knockdown caused a decrease in GBM cell proliferation, colony formation, invasion, and migration and induced G2/M cell-cycle arrest. High PSMD9 expression is associated with reduced sensitivity to panobinostat (HDAC inhibitor). Functionally, PSMD9 overexpression rescues panobinostat-induced growth inhibition, cell-cycle arrest, and anti-invasive effects, both in vitro and in vivo [66]. Moreover, PSMD9 knockout in MCF7 breast cancer cells resulted in smaller colony size, reduced anchorage-independent growth, and increased sensitivity to bortezomib [37].

PSMD9 is also a prognostic biomarker in hepatocellular carcinoma (HCC), with high expression linked to poor outcomes. It promotes HCC proliferation, migration, and metastasis. PSMD9 silencing inhibits tumor growth by inducing cell cycle arrest and apoptosis. PSMD9 knockdown sensitizes HCC cells to erlotinib, emphasizing its oncogenic role as a protein-protein interaction mediator [31].

Although several independent datasets report elevated PSMD9 levels in specific cancers, its biomarker potential remains preliminary. This is due to variability in cohort sizes, reliance on semi-quantitative IHC, and limited prospective validation. Therefore, further confirmation using orthogonal quantification methods (qPCR/proteomics) and multi-center clinical cohorts is needed to confirm PSMD9 as a biomarker for disease progression and therapy resistance. Moreover, despite growing functional evidence linking PSMD9 to therapy resistance, whether these effects are mediated through proteasome-dependent mechanisms or arise from proteasome-independent protein–protein interactions remains unresolved, underscoring an important gap in our mechanistic understanding.

### Domain-motif interactions of PSMD9, designer peptides, and inhibitory potency

**Figure 4:**
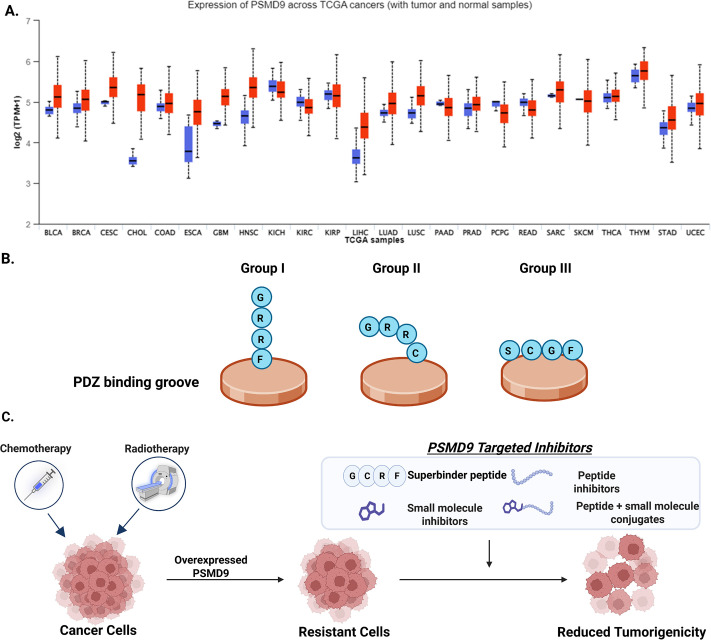
PSMD9 as a potential therapeutic target

PSMD9 is overexpressed in many cancers (Figure 4) and is associated with therapy resistance or tumor progression in some cancers. PSMD9’s multi-domain structure enables diverse protein-protein interactions. Knowledge about the structure and fold of the domains and their specific interaction can pave the way for the design of inhibitors. Using structural bioinformatics approaches, our studies demonstrated that the PDZ domain of PSMD9 binds C-terminal motifs in various proteins, including those involved in RNA processing and signaling [67]. We identified three distinct interactions of the PDZ domain of PSMD9, redefining its ability to interact with cellular proteins (Figure 4). We found that the peptide that belongs to Group I, such as the GRRF, binds with moderate affinity (666 μM), while GRRG showed no binding. Here, the P0 residue is important, as known for other PDZ domain interactions. GRRF inhibited PSMD9’s interaction with hnRNPA1 by 80%, but GRRG was ineffective. Group II peptides bound the domain with moderate affinity, and additionally, high-affinity peptides (~10 μM) sensitive to P-2 substitutions, like SCGF (Group III peptides), inhibited 90-95% of the PSMD9-hnRNPA1 interaction. Successive iterations of C-terminal motifs led to the creation of super binding peptides GCRF and GCRG (~5 μM), which also inhibited ~ 95% of the interaction [68]. This study defines a unique PDZ motif with a strong preference for cysteine residue at P-2 and a hydrophobic residue at P0 position, establishing a scaffold for designing peptide-based inhibitors to block NF-kB signaling in cancer (Figure 4).

(**A**) Box plots show PSMD9 mRNA expression across multiple TCGA cancer types, comparing tumor (red) and matched normal (blue) samples, highlighting frequent overexpression of PSMD9 in several malignancies.Tumor type abbreviations are as follows: BLCA (bladder urothelial carcinoma), BRCA (breast invasive carcinoma), CESC (cervical squamous cell carcinoma and endocervical adenocarcinoma), CHOL (cholangiocarcinoma), COAD (colon adenocarcinoma), ESCA (esophageal carcinoma), GBM (glioblastoma multiforme), HNSC (head and neck squamous cell carcinoma), KICH (kidney chromophobe), KIRC (kidney renal clear cell carcinoma), KIRP (kidney renal papillary cell carcinoma), LIHC (liver hepatocellular carcinoma), LUAD (lung adenocarcinoma), LUSC (lung squamous cell carcinoma), PAAD (pancreatic adenocarcinoma), PRAD (prostate adenocarcinoma), PCPG (pheochromocytoma and paraganglioma), READ (rectum adenocarcinoma), SARC (sarcoma), SKCM (skin cutaneous melanoma), THCA (thyroid carcinoma), THYM (thymoma), STAD (stomach adenocarcinoma), and UCEC (uterine corpus endometrial carcinoma). (**B**) Peptide optimization strategy against PSMD9 exploiting the peptide binding groove in the PDZ domain. Tested peptide examples: GRRF (low affinity), GRRC (high affinity), and SCGF (tight binder) with probable binding modes of perpendicular, intermediate, and canonical orientation, respectively. (**C**) Design principles in PSMD9 domain-motif interaction can be used to generate peptide inhibitors of PSMD9.SLiM-based peptide inhibitors, i.e., GCRF, could be used as a strategy either alone or in combination with inhibitors of proteasome, other pathways, and/or organelle function where PSMD9 overexpression is accompanied by therapy resistance.

Besides the PDZ domain- C terminal motif, we identified a conserved EXKK in 56 PSMD9-associated proteins [55], and this motif bound specifically to the N-terminal coiled-coil domain of PSMD9. EXKK (corresponding notations P-3P-2P-1P0) is a molecular mimic of the QAKK present in PSMC3, the ATPase that binds PSMD9 in the assembly process. Incidentally, among the interactors, after Glu (E), the next residue enriched at the P-3 position was Gln (Q). This discovery and analysis highlight how cancer cells can exploit existing protein interaction principles to expand the repertoire of interactions by proteins whose levels are altered in cancer cells, where mutations are not common. Indeed, PSMD9 knockout in MCF7 cells significantly reduces tumorigenicity, proposing that targeting PSMD9-regulated pathways could be catastrophic for cancer.

To conclude, this article highlights the emerging role of PSMD9 as more than a mere proteasome assembly chaperone, positioning it instead as a regulatory node that links proteasome biogenesis with cellular stress response pathways. By modulating proteasome architecture and subcomplex stoichiometry, PSMD9 regulates the targeted degradation of essential signaling proteins, thereby maintaining proteostasis during metabolic, proteotoxic, and genotoxic stress conditions. Elevated PSMD9 levels may allow cancer cells to exploit adaptive proteasome states, reconfigure protein–protein interaction networks, and withstand therapeutic stress, thereby contributing to resistance to radiation and other treatments.

These observations advocate for transitioning from focusing on proteasome catalytic activity to instead targeting the disruption of assembly processes and regulatory interfaces. The C-terminal PDZ and N-terminal coiled-coil domains of PSMD9 present potential avenues for interaction-specific targeted interventions. More broadly, PSMD9 exemplifies a class of stress-responsive proteasomal chaperones whose emerging functions probably indicate an underexplored dimension of proteostasis regulation with significant implications for cancer susceptibility and therapeutic resistance.

**Summary**

- PSMD9 serves as a critical chaperone orchestrating proteasome assembly and modulating the stoichiometry of its 20S and 26S/30S complexes.
- Its depletion impairs proteasome function, triggering proteotoxic and oxidative stress while destabilizing organelle structures, particularly the nucleolus and mitochondria.
- Through specific domain interactions, PSMD9 integrates protein homeostasis with crucial pathways like translation, lipid metabolism, and the unfolded protein response.
- PSMD9 expression correlates and associates with therapy resistance in several cancers, implying that PSMD9 could be a key therapeutic target.

## Open Access

This article has been published open access under our Subscribe to Open programme, made possible through the support of our subscribing institutions, learn more here: https://portlandpress.com/pages/open_access_options_and_prices#conditional

## Abbreviations

HCC: hepatocellular carcinoma
SLiM: short linear motif
UPS: ubiquitin-proteasome system
